# C5aR1 is a master regulator in Colorectal Tumorigenesis via Immune modulation

**DOI:** 10.7150/thno.45058

**Published:** 2020-07-09

**Authors:** Peipei Ding, Ling Li, Luying Li, Xinyue Lv, Danlei Zhou, Qingkai Wang, Jianfeng Chen, Chaoqun Yang, Enjie Xu, Weixing Dai, Xin Zhang, Na Wang, Qi Wang, Wei Zhang, Long Zhang, Yuzhen Zhou, Hongyu Gu, Qunying Lei, Xuhui Zhou, Weiguo Hu

**Affiliations:** 1 Fudan University Shanghai Cancer Center and Institutes of Biomedical Sciences, Shanghai Medical College, Fudan University, Shanghai 200032, China.; 2 Changzheng Hospital, Second Military Medical University, Shanghai 200003, China.; 3 Department of Colorectal Surgery, Fudan University Shanghai Cancer Center, Shanghai 200032, China.; 4 Key Laboratory of Breast Cancer in Shanghai, Fudan University Shanghai Cancer Center, Fudan University, Shanghai, China.

**Keywords:** Complement, C5aR1, colorectal tumorigenesis, immune modulation

## Abstract

Numerous factors have been claimed to play important roles in colorectal cancer (CRC) tumorigenesis, including myeloid-derived suppressor cells (MDSCs) and other immune cells, cytokines, and chemokines; however, the precise mechanisms of colorectal tumorigenesis remain elusive, and there is a lack of effective preventive treatments. Here, we investigated the role of complement system, a key regulator of immune surveillance and homeostasis, in colorectal tumorigenesis.

**Methods:** The prototypical CRC model was induced by combined administration of azoxymethane (AOM)/ dextran sulfate sodium (DSS) in Wild-type (WT), *C3*-, *C5*-, *C5ar1*-, and *C5ar2*-deficient mice. Using flow cytometry, immunohistochemical staining and multiplex bead assay, we profiled the immune cells, cytokines and chemokines. Bone marrow transplantation was employed to determine the contribution of immune cells in colorectal tumorigenesis. Further, we used C5aR1 antagonist PMX205 to investigate the protective role in colorectal tumorigenesis.

**Results:** Complement was extensively activated in inflamed tissues of AOM/DSS-induced murine CRC model, leading to multifaceted consequences. The deficiency of complement *C5* or especially *C5ar1*, but not *C3* almost completely prevented CRC tumorigenesis. C5a/C5aR1 signaling recruited MDSCs into the inflamed colorectum to impair CD8^+^ T cells, and modulated the production of critical cytokines and chemokines, thus initiating CRC. Moreover, the C5aR1 antagonist PMX205 strongly impeded colorectal tumorigenesis. Bone marrow transplantation further revealed that C5aR1 expression by immune cells was critical for colorectal tumorigenesis.

**Conclusion:** Our study identifies C5a/C5aR1 signaling as a vital immunomodulatory program in CRC tumorigenesis and suggests a feasible preventive strategy.

## Introduction

Colorectal cancer (CRC) ranks 3^rd^ in terms of cancer morbidity and mortality 1; however, the molecular mechanisms that underlie CRC pathogenesis still remain obscure. In addition to the inherited genetic disorders and epigenetic changes, the epidemiologic and experimental evidence strongly implicates that chronic inflammation not only tightly links inflammatory bowel disease (IBD) and colitis-associated cancer (CAC) but is strongly associated with other types of CRC. Thus, chronic inflammation is recognized as a critical factor in virtually all steps of CRC development, including initiation, promotion, progression, and metastasis 2. At sites of chronic colorectal inflammation, immune cells such as myeloid-derived suppressor cells (MDSCs) are suspected to promote tumorigenesis by shaping a tumor-promoting immunosuppressive microenvironment 3, 4. Moreover, pro-inflammatory cytokines such as TNF-α, IL-1 and IL-6 can activate oncogenic signaling pathways 5, 6, and chemokines and/or their receptors, such as CCL2 3 and CXCR2 4, can initiate CRC by inducing the recruitment and development of MDSCs. Therefore, colorectal tumorigenesis appears to involve multifaceted immune regulation.

The complement system has been recognized as a prominent arsenal of innate immunity and a bridge to adaptive immunity for immune surveillance and homeostasis 7-9. Complement executes versatile functions through its various enzymatic products, including direct lysis of pathogens by membrane attack complex (MAC), opsonization of pathogenic substances by C3b/iC3b, and induction of anaphylactic reactions by C3a/C5a. Accumulating evidence also shows that complement emerges as a key regulator of cancer immunity, thus involving, to varying degree, in tumor initiation and development 10-14. Intratumoral C5a recruits MDSCs to tumor microenvironment by binding to its receptor C5aR1 on MDSCs, further inhibiting cytotoxic T cell function and promoting xenograft tumor growth 15. Deficiency of the complement regulatory protein PTX3 increases susceptibility to skin tumorigenesis by inducing excessive complement activation and further enhances macrophage infiltration, cytokine production, and angiogenesis 16. Anaphylatoxin C5a, as a potent inflammatory mediator, was recently revealed to cultivate an immunosuppressive microenvironment by activating C5aR1^+^ mast cells and macrophages, thus fostering squamous carcinogenesis 17.

The C3a serum level significantly elevates in CRC patients, with high sensitivity and specificity 18, indicating excessive complement activation involved in CRC development. Complement depletion restricts tumor growth in a transplantable murine model of colon cancer 19. Moreover, *in vitro* studies demonstrate that anaphylatoxin C5a enhances human colon cancer cell motility and invasiveness via aberrantly expressed C5aR1 on these tumor cells 20. Further, *in vivo* studies show that C5a augments tumor metastasis of colon cancer via stimulating M2 polarization of tumor-associated macrophages (TAMs) by interacting with C5aR1 21, 22.

Although complement effect especially on CRC progression has been extensively investigated, there are very limited studies to address the implication in colorectal tumorigenesis 10-14. In an *Apc*^Min/+^ based and high fat diet-induced mouse model of intestinal neoplasia, complement is activated and its active fragment C5a acts as the trigger for inflammation and intestinal tumorigenesis 23. In addition, in a colitis-induced colorectal cancer model, complement activation promotes CRC tumorigenesis via activating intestinal IL-1β/IL-17A axis yet in an uncertain mouse genetic background 24. Therefore, the diverse roles of different complement components in colorectal tumorigenesis still require further exploration for the feasible preventive strategies.

Here, we induced a prototypical CAC model by combined administration of azoxymethane (AOM) and dextran sulfate sodium (DSS) in the C57BL/6 genetic background mice with deficiency of *C3*, *C5*, *C5ar1* or *C5ar2*, and revealed that C5aR1 signaling, independent of C3 activation, plays a key role in the colitis-associated colorectal tumorigenesis by modulating the cellular and molecular immune responses. Our finding suggests that blockade of C5aR1 signaling may represent a promising approach to preventing colorectal tumorigenesis.

## Materials and Methods

### Reagents and antibodies

The commercial antibodies used in this study were shown in Table S1. The C5aR1 antagonist PMX205 was purchased from Tocris Bioscience (R&D Systems, Minneapolis, MN).

### Mice

Eight to twelve weeks-old male mice were used for all experiments. C57BL/6 Wild-type (WT) mice were obtained from SLAC Laboratory Animal Co., Ltd. (Shanghai, China). Mice deficient in *C3*, *C5* 25 or *C5ar1* were purchased from Jackson Laboratory (Bar Harbor, ME), and *C5ar2-*deficient mice were purchased from Taconic (Rensselaer, NY); all these mice were backcrossed onto the C57BL/6 genetic background for over 10 generations. All mice were housed under specific pathogen-free conditions at the animal facility of Shanghai Medical School, Fudan University. All animal care and experimental procedures were approved by the Animal Ethics Committee at Shanghai Medical School, Fudan University, in compliance with the Guide for the Care and Use of Laboratory Animals published by the US National Institutes of Health.

### Induction of murine CRC by AOM/DSS

Experimental murine colitis-associated tumorigenesis was induced as described previously 26, with slight modifications. Briefly, the mice were intraperitoneally administered the mutagen azoxymethane (AOM, 8.5 mg/kg body weight, Sigma-Aldrich, St. Louis, MO) on Day 1 and 8. One week later, these mice received 1.5% (w/v) the proinflammatory agent dextran sodium sulfate (DSS, MW 36-50kDa; MP Biomedical, Solon, OH) in their drinking water ad libitum for 7 days, followed by 14 days of regular water. Then, the mice were subjected to two more DSS treatment cycles. The clinical disease score was monitored continuously in terms of body weight, stool consistency and the presence of blood in stool during DSS treatment. For the intervention of AOM/DSS-induced CRC, the C5aR1 antagonist, PMX205 was given by gavage every other day after two AOM injections until the endpoint of experiment.

### Generation of bone marrow (BM) chimeric mice

BM cells were collected from 6- to 8-week-old female donor WT and *C5ar1*-deficient mice by flushing the femurs and tibias with PBS. Recipient male mice were lethally irradiated with 9.0 Gy γ-rays (2 doses of 4.5 Gy with a 3 h resting interval). Then, the recipient irradiated mice received 1.0×10^7^ donor BM cells intravenously. After 4 to 6 weeks, the BM in chimeric mice was reconstituted, and subsequent experimental procedures were performed.

### Histology and immunohistochemical (IHC) staining

Briefly, colon segments from mice were fixed overnight in 10% formalin, embedded in paraffin blocks, cut into 3-5 μm sections, and stained with hematoxylin and eosin (H&E), as described previously 27.

For IHC staining, colon sections were incubated with 3% hydrogen peroxide to block endogenous peroxidase activity for 15 min at 37°C, followed by high-pressure antigen retrieval in citrate buffer (pH 6.0). Then, mouse colon sections were incubated with an affinity purified pAb specific for mouse complement component C3d (1:80; R&D Systems, Minneapolis, MN), rabbit anti-Ki67 antibody (1 μg/ml; Abcam, Cambridge, MA) or rabbit anti-CD8 alpha antibody (1:1500; Abcam, Cambridge, MA) at 4°C overnight. After 3 rinses in PBS, the sections were incubated with HRP-conjugated rabbit anti-goat IgG H&L (1:200; Abcam, Cambridge, MA) or HRP-conjugated goat anti-rabbit IgG H&L (1:400; Abcam, Cambridge, MA) at room temperature (RT) for 1 h. Immunoreactivity was detected using a GTVision III immunohistochemical detection kit (GK500705; Gene Tech, Shanghai, China) according to the manufacturer's instructions. Isotype IgG controls were included according to the same procedure as primary Abs.

For the quantification of CD8^+^ T cells infiltrating into the colons, the numbers of CD8 alpha staining positive cells were counted by two experienced pathologists independently using the Olympus BX53 microscope (Olympus, Tokyo, Japan). In each specimen, 10 randomly selected high-power fields (HPF, magnification, X400), including both tumor and mucosa-associated lymphoid tissue (MALT) areas were assessed and the results were averaged.

### Enzyme-linked immunosorbent assay (ELISA)

The level of mouse C5a in colon tissue homogenates (CTHs) was measured with Mouse Complement Component C5a DuoSet ELISA kit according to the manufacturer's instructions (R&D Systems, Minneapolis, MN).

### Flow cytometry

For leukocyte immunostaining, mouse blood, spleen and colon samples were obtained to prepare single cell suspensions. The fixable viability dye eFluor® 780 (eBioscience, Thermo Fisher Scientific, MA) was used to label dead cells. To block nonspecific binding of Fc receptors, cells were pre-incubated with a purified anti-CD16/32 antibody for 10 min on ice prior to immunostaining. Then, the cells were stained with the appropriate combination of the following fluorochrome-conjugated mAbs against mouse CD11b (FITC), CD3ε (FITC), CD8α (PE), NK1.1 (PE), F4/80 (PE), Gr-1 (APC), CD19 (APC), CD4 (PE/CY7) or C5aR-1 (PE) (Biolegend, San Diego, CA) on ice for 30 min according to the manufacturer's instructions, in which the fluorochrome-conjugated isotype-specific IgGs were treated as controls to determine the quadrants and gates. All samples were assayed by a BD FACSCanto^TM^ II Cell Analyzer (BD Biosciences, San Jose, CA), and the data were analyzed with FlowJo software (Tree Star, Ashland, OR).

### Profiling of cytokines and chemokines

Briefly, harvested colon samples were homogenized in T-PER tissue protein extraction lysis buffer (Pierce, Thermo Fisher Scientific, MA) supplemented with protease inhibitor cocktail (Biotool, Houston, TX), and the supernatants were collected. Then, the local levels of inflammatory mediators in CTHs were measured by using LEGENDplex^TM^ Multiplex Bead Assays (Biolegend, San Diego, CA), of which the Mouse Proinflammatory Chemokine Panel (Cat. No. 740007, 13-plex for CCL2/MCP-1, CCL3/MIP-1α, CCL4/MIP-1β, CCL5/RANTES, CCL11/Eotaxin, CCL17/TARC, CCL20/ MIP-3α, CCL22/MDC, CXCL1/KC, CXCL5/LIX, CXCL9/MIG, CXCL10/IP-10 and CXCL13/BLC), Mouse T Helper Cytokine Panel (Cat. No. 740005, 13-plex for IL-2, IL-4, IL-5, IL-6, IL-9, IL-10, IL-13, IL-17A, IL-17F, IL-21, IL-22, IFN-γ and TNF-α) and Mouse Cytokine Panel 2 (Cat. No. 740134, 13-plex for IL-1α, IL-1β, IL-3, IL-7, IL-11, IL-12p40, IL-12p70, IL-23, IL-27, IL-33, IFN-β, GM-CSF and TSLP) were selected. Cytokines/chemokines was profiled on a BD FACSCanto^TM^ II Cell Analyzer (BD Biosciences, San Jose, CA), and the FCS file generated was analyzed by using Biolegend's LEGENDplex^TM^ Data Analysis Software according to the manufacturer's instructions.

### Statistical analysis

All statistical analyses were carried out using GraphPad Prism 7.0 (GraphPad Software, La Jolla, CA). Data are presented as the mean ± SEM or Min to Max. Differences in parametric data between two groups were evaluated by Student's* t*-test (two-tailed). For correlation analysis between groups, Pearson's correlation coefficient test was used if the data fit a normal distribution; otherwise, Spearman's rank correlation coefficient test was applied. The cumulative survival time was calculated using the Kaplan-Meier method, and the differences were analyzed using the log-rank test. The significance of body weight changes was determined by two-way ANOVA. Values of *P* <0.05 were regarded as statistically significant, and significance is presented as * *P* <0.05, ** *P* <0.01, *** *P* <0.001, and **** *P* <0.0001.

## Results

### Deficiency of *C5* or *C5ar1* prevents AOM/DSS-induced CRC

**Figure 1:**
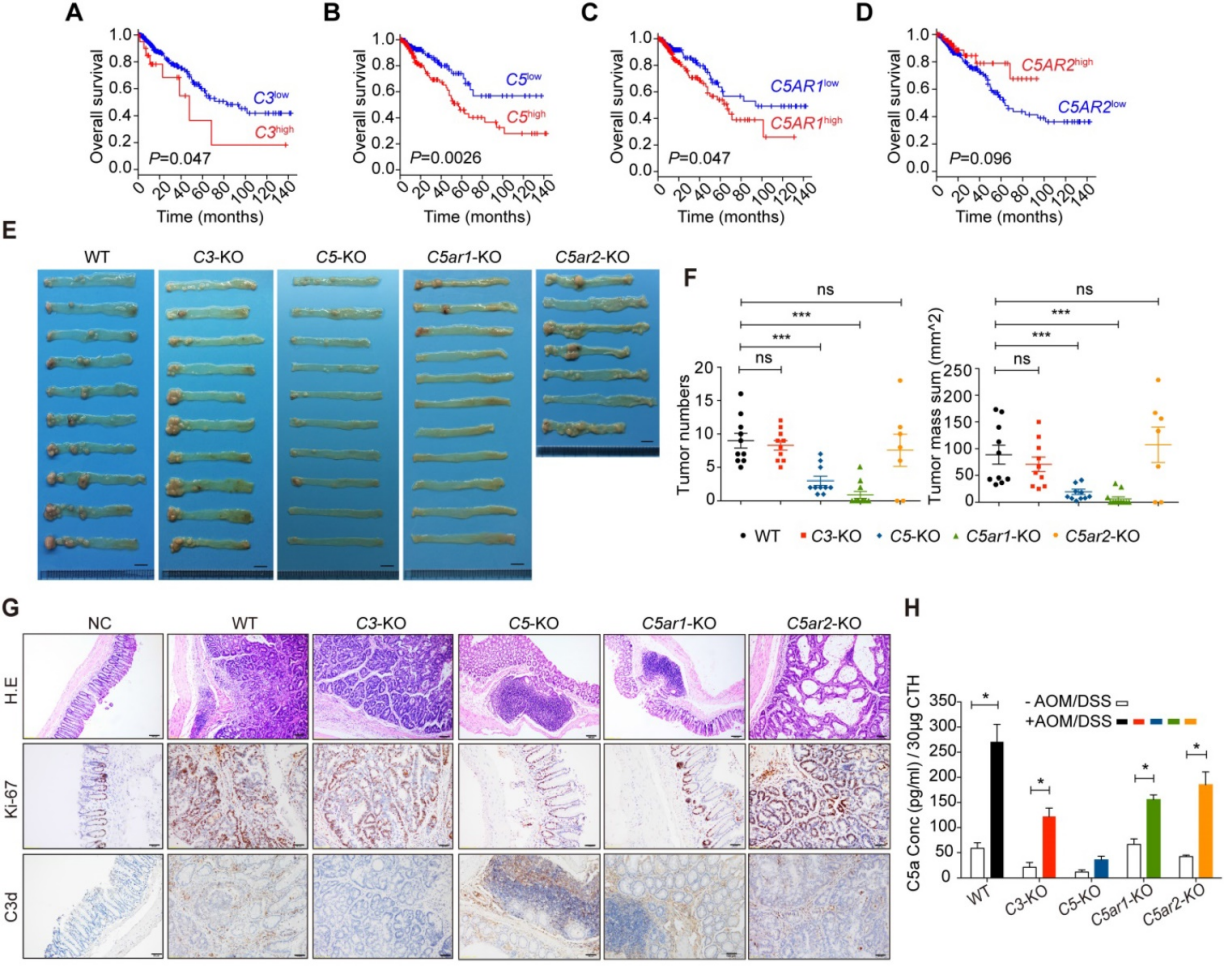
**The strong correlation between complement activation and CRC.** (**A-D**) The associations between the mRNA levels of *C3* (A), *C5* (B), *C5AR1* (C), and *C5AR2* (D) and the overall survival of CRC patients (n=364). (**E, F**) The effect of *C3*, *C5*, *C5ar1* or *C5ar2* deficiency on AOM/DSS-induced colorectal tumorigenesis compared to WT control. Images of the colorectum, where CRC often developed at distal (rectum) sites (E), and quantitative analysis of tumor number and mass (F). The experiment was duplicated. Scale bar in (E), 1 cm. (**G**) Pathological analysis of control or tumor tissues, including H&E, Ki-67 and C3d staining. Scale bar, 100 µm for H&E and 50 µm for IHC. (**H**) C5a concentration in colon tissue homogenates (CTHs) measured by ELISA. n≥ 7 in each group of WT, *C3*-KO, *C5*-KO, *C5ar1*-KO, or *C5ar2*-KO mice. Data are represented as mean ± SEM; ns, not significant; * *P*<0.05; and *** *P*<0.001. NC, negative control.

C3 and C5 are critical components of the middle and late stages of complement activation, respectively, and C5aR1 and C5aR2, which may have opposite functions 28, are two receptors for the potent pro-inflammatory mediator C5a. We performed Kaplan-Meier analysis of a TCGA cohort of 364 CRC patients (Table S2) and found that high expression of *C3*, *C5*, and *C5AR1* but not of *C5AR2* significantly predicted poor overall survival (Figure 1A to D), indicating the potential role of complement activation during CRC progression.

To further investigate the role of complement activation in CRC initiation, we induced CRC by administering AOM and DSS to *C3*-, *C5*-, *C5ar1-*, and *C5ar2*-deficient mice (Figure S1A) 26. Compared to WT mice,* C5ar2*-deficient mice showed more severe DSS-induced inflammation, while mice deficient in *C3*, *C5*, or* C5ar1* showed attenuated disease, as indicated by dynamic survival rate and body weight loss in mice (Figure S1B-C). More importantly, *C5* and especially *C5ar1* deficiency almost completely prevented tumorigenesis (Figure 1E), as determined by the quantification of tumor numbers and size (Figure 1F). H&E staining showed that adenoma formation was accompanied by diffuse immune cells infiltration in the colons of WT, *C3*-deficient, and *C5ar2*-deficient mice, while more mucosa-associated lymphoid tissues suspected as tumors (as shown in Figure 1E) were observed in *C5*- and *C5ar1*-deficient mouse colons (Figure 1G, upper panel). The strong staining for Ki-67 in WT, *C3*-deficient, and *C5ar2*-deficient, but not *C5*-deficient and *C5ar1*-deficient mouse colons indicated that intensive cell proliferation occurred only in the presence of C5 and C5aR1 (Figure 1G, middle panel). The significant increases in local complement cleavage products, including C3d (Figure 1G, lower panel) and C5a (Figure 1H), revealed comprehensive complement activation at inflamed colorectal sites. Therefore, these results suggest that complement activation may be highly involved in the process of AOM/DSS-induced colorectal tumorigenesis and, more importantly, that C5a/C5aR1 but not C3 may play a critical role.

### Loss of C5 or C5aR1 diminishes a massive infiltration of MDSCs and elevates CD8^+^ T cell proportion in mice upon AOM/DSS treatment

**Figure 2:**
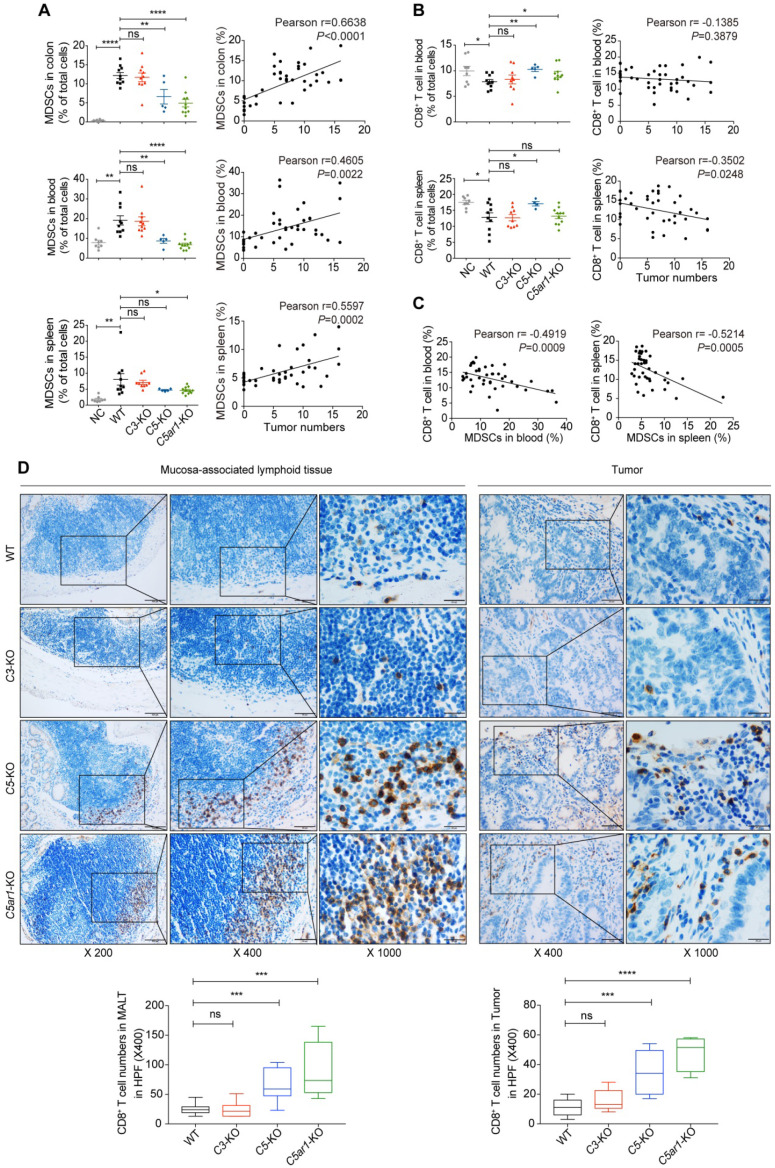
** Loss of C5 or C5aR1 notably inhibits MDSCs infiltration and elevates CD8^+^ T cells proportion in AOM/DSS-induced CRC.** (**A, B**) Flow cytometry analysis of MDSC (A) and CD8^+^ T cell (B) proportions in the indicated tissues of WT, *C3*-deficient, *C5*-deficient, and *C5ar1*-deficient mice upon AOM/DSS treatment compared to those in WT mice without AOM/DSS treatment (NC) (left panel) and their correlation with tumor number (data in all groups were pooled for the analysis, right panel). (**C**) The correlation between the proportions of MDSCs and CD8^+^ T cell in the spleen and blood. (**D**) Representative immunohistochemistry staining of CD8^+^ T cell in colons of AOM/DSS-treated WT, *C3*-deficient, *C5*-deficient, and *C5ar1*-deficient mice. Left panel, mucosa-associated lymphoid tissue (MALT) (Scale bar, 100 µm, 50 µm, 20 µm); Right panel, tumor or counterpart tissue (Scale bar, 50 µm, 20 µm). Quantification of the numbers of CD8^+^ T cell shown on the bottom. Data are represented as mean ± SEM or Min to Max; n≥5 in each group; ns, not significant; * *P*<0.05; ** *P*<0.01; *** *P*<0.001; and **** *P*<0.0001.

To decipher the underlying mechanisms by which C5a/C5aR1 signaling promotes colorectal tumorigenesis, we first investigated the cellular immune response. Colon tumors (or counterpart tissues in *C5*- or *C5ar1*-deficient mice), spleen and blood samples were collected from mice for profiling immunocytes by flow cytometry (Figure S2) or IHC assay. The proportion of MDSCs, defined as CD11b^+^Gr-1^+^, in the tested tissues from WT mice was potently elevated upon AOM/DSS treatment (Figure 2A, left panel), and this effect was remarkably reduced by loss of C5 or C5aR1 but not of C3 (Figure 2A, left panel). More importantly, the proportion of MDSCs in these tissues was highly positively correlated with tumor numbers (Figure 2A, right panel). In contrast, the proportion of CD8^+^ T cells in the blood and spleen of WT mice was significantly reduced upon AOM/DSS treatment, and this effect was reversed by deficiency in *C5* or *C5ar1* (blood only) but not in *C3* (Figure 2B, left panel). Furthermore, the proportion of CD8^+^ T cells in the spleen, but not in the blood, was inversely correlated with tumor numbers (Figure 2B, right panel). Notably, the MDSC proportion was negatively associated with the CD8^+^ T cell proportion in the blood and spleen (Figure 2C). To detect the accumulation of CD8^+^ T cells in the colons after AOM/DSS treatment, we performed IHC staining due to its low abundance for flow cytometry assay. We found that there were much more CD8^+^ T cells in mucosa-associated lymphoid tissues of *C5*- or *C5ar1*-deficient mice than those of WT or *C3*-deficient mice (Figure 2D left panel). In consistent, the counterpart tissues of *C5-* and *C5ar1*-deficient mice were infiltrated with significantly greater numbers of CD8^+^ T cells compared with the tumor tissues of WT and *C3-*deficient mice (Figure 2D right panel). These results suggest that MDSCs may contribute to CD8^+^ T cell suppression 4, 15, 29, 30, thus shaping a pro-tumor immunosuppressive microenvironment.

In addition, the proportion of macrophages increased and the proportions of B, CD4^+^ T and NK cells decreased in the blood and spleen of WT mice upon AOM/DSS treatment (Figure S3A to D). Deficiency in *C5* and especially in *C5ar1*, but not in *C3,* could reverse these changes in macrophage and B cell proportions to varying degrees (Figure S3A-B). However, compared to that in WT control mice, the proportion of NK cells was unexpectedly further decreased in the blood and spleen of *C5*- and *C5ar1*-deficient mice (Figure S3D), indicating that NK cells are likely unrelated to C5- and C5aR1-mediated CRC prevention. Taken together, these findings suggest that local C5a may recruit MDSCs by binding to C5aR1, thus impairing CD8^+^ T cell propagation and function and ultimately promoting AOM/DSS-induced colorectal tumorigenesis.

### Loss of C5 or C5aR1 modifies local levels of various cytokines and chemokines in mice treated with AOM/DSS

**Figure 3:**
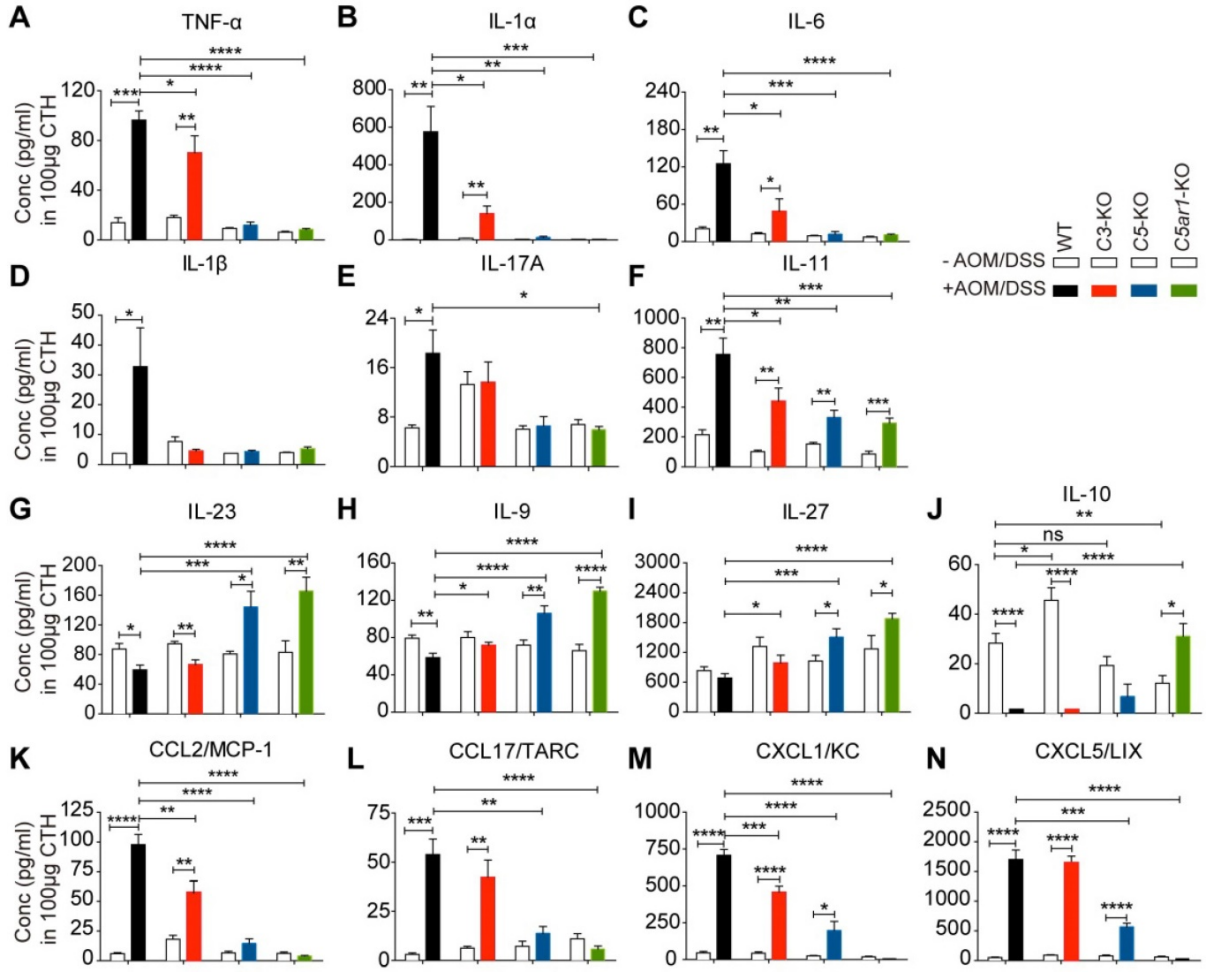
** The profiles of multiple cytokines/chemokines in AOM/DSS-induced CRC mice.** (**A-F**) The effect of *C3*, *C5*, and *C5ar1* deficiency on the local levels of the proinflammatory cytokines TNF-α (A), IL-1α (B), IL-6 (C), IL-1β (D), IL-17A (E) and IL-11 (F). (**G-N**) Local levels of the anti-inflammatory cytokines IL-23 (G), IL-9 (H), IL-27 (I) and IL-10 (J); and the chemokines CCL2 (K), CCL17 (L), CXCL1 (M) and CXCL5 (N) in the related mice upon AOM/DSS treatment. Data are represented as mean ± SEM; n≥5 in each group; ns, not significant; * *P*<0.05; **P<0.01; *** *P*<0.001; and **** *P*<0.0001.

Accumulating evidence has suggested that chronic colitis promotes tumorigenesis mainly by altering the production of a variety of cytokines/chemokines, such as TNF-α, IL-1/6/10, TGF-β, CCL2/4, and CXCL1/2/5 3, 4, 31, 32. Thus, we profiled the levels of 39 cytokines/chemokines in colon tissue homogenates (CTHs) from WT and *C3*-, *C5*- and *C5ar1*-deficient mice with or without AOM/DSS treatment. AOM/DSS treatment remarkably increased local TNF-α levels in WT and *C3*-deficient mice but not in *C5*- or *C5ar1*-deficient mice (Figure 3A). Moreover, upon AOM/DSS treatment, TNF-α levels were much lower in *C5*- and *C5ar1*-deficient mice, and to a lesser extent in *C3*-deficient mice, than in WT mice (Figure 3A). Similar patterns were also observed for the levels of IL-1α (Figure 3B), IL-6 (Figure 3C), IL-1β (Figure 3D), and IL-17A (Figure 3E), with the exception that AOM/DSS treatment did not increase IL-1β or IL-17A levels in *C3*-deficient mice. In addition, AOM/DSS treatment significantly increased IL-11 levels in all four groups of mice; however, deficiency in *C3*, *C5*, or *C5ar1* significantly reduced IL-11 levels compared to WT control (Figure 3F).

In contrast, while WT and *C3*-deficient mice showed reduced levels of anti-inflammatory cytokines to varying degrees, those deficient in *C5* or *C5ar1* instead showed significantly elevated levels of IL-23 (Figure 3G), IL-9 (Figure 3H) and IL-27 (Figure 3I) upon AOM/DSS treatment compared to WT mice. Notably, IL-10 levels under physiological conditions were significantly elevated in *C3*-deficient mice and reduced in *C5ar1*-deficient mice compared to WT mice (Figure 3J). However, upon AOM/DSS treatment, IL-10 was reduced to an almost undetectable level in WT and *C3*-deficient mice but was significantly elevated in* C5ar1*-deficient mice (Figure 3J), indicating that IL-10 may play a unique role in AOM/DSS-induced CRC in mice. The local levels of the other three detectable cytokines, IL-12, IFN-γ and IL-2, failed to show correlations with tumor development (Figure S4A to C).

In addition, the local levels of four chemokines, CCL2, CCL17, CXCL1 and CXCL5, were positively correlated with tumorigenesis. The levels of these chemokines remained very low in the normal colorectum in the four groups of mice but increased remarkably in WT and *C3*-deficient mice upon AOM/DSS treatment (Figure 3K to N). Although the levels of CXCL1 and CXCL5, but not of CCL2 and CCL17, significantly increased upon AOM/DSS treatment in *C5*-deficient mice, they were still much lower than those in WT mice (Figure 3K to N). Notably, the levels of the above four chemokines remained unchanged in *C5ar1*-deficient mice regardless of AOM/DSS treatment (Figure 3K to N). Although the levels of CCL22, a CCR4 ligand similar to CCL17, increased upon AOM/DSS treatment, they were still significantly lower in AOM/DSS-treated *C5*- and *C5ar1*-deficient mice than in WT mice (Figure S4D). The levels of the other eight detectable chemokines, CCL3/4/5/11/20 and CXCL9/10/13, were not highly correlated with the pattern of CRC development in the four groups of mice (Figure S4E to L). Together, these findings suggest that C5a/C5aR1 signaling may also modulate the local production of multiple pro- and anti-inflammatory cytokines and chemokines to foster AOM/DSS-induced CRC in mice.

### A C5aR1 antagonist, PMX205, impedes AOM/DSS-induced CRC

**Figure 4:**
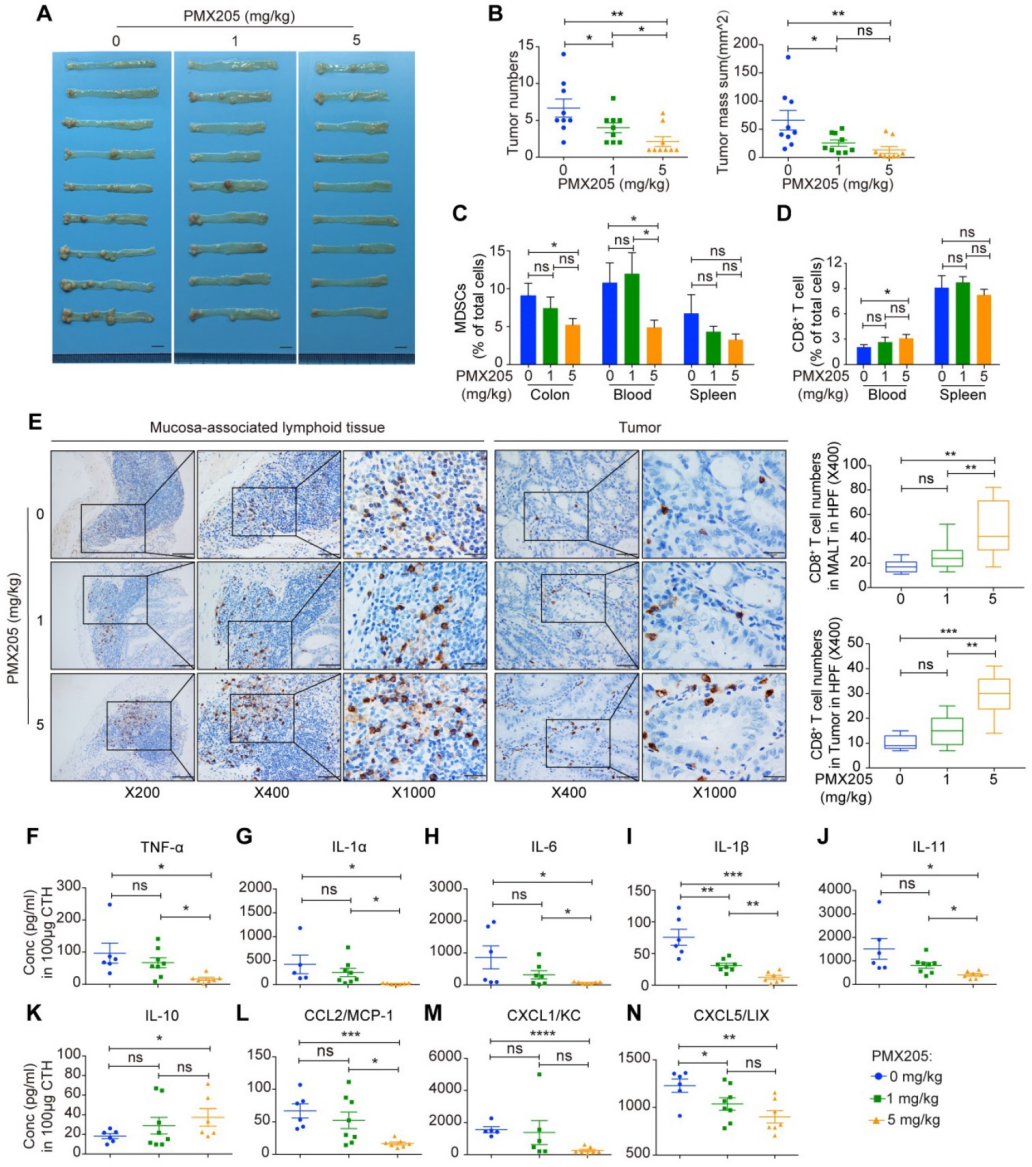
** A C5aR1 antagonist, PMX205, impedes AOM/DSS-induced CRC tumorigenesis.** (**A, B**) The effect of PMX205 treatment on AOM/DSS-induced CRC in mice. Images of the colorectum (A) and quantitative analysis of tumor number and mass (B). Scale bar, 1 cm. (**C, D**) Flow cytometry analysis of MDSCs (C) and CD8^+^ T cell (D) proportions in the indicated tissues of AOM/DSS-treated mice with or without PMX205 administration. (**E**) Representative immunohistochemistry staining of CD8^+^ T cell in colons. Left panel, mucosa-associated lymphoid tissue (MALT) (Scale bar, 100 µm, 50 µm, 20 µm); Right panel, tumor tissue (Scale bar, 50 µm, 20 µm). Quantification of the numbers of CD8^+^ T cell shown on the right. (**F-N**) Local levels of the proinflammatory cytokines TNF-α (F), IL-1α (G), IL-6 (H), IL-1β (I) and IL-11 (J); the anti-inflammatory cytokine IL-10 (K); and the chemokines CCL2 (L), CXCL1 (M) and CXCL5 (N). Data are represented as mean ± SEM or Min to Max; n≥5 in each group; ns, not significant; * *P*<0.05; ** *P*<0.01; *** *P*<0.001; and **** *P*<0.0001.

Based on our above finding that loss of C5aR1 potently prevented AOM/DSS-induced CRC, we next employed a C5aR1 antagonist, PMX205, to assess its impact on this malignant transformation. Indeed, treatment with the C5aR1 antagonist PMX205 radically impeded AOM/DSS-induced mouse CRC in a dose-dependent manner (Figure 4A-B).

Furthermore, in line with the previous results obtained in *C5ar1*-deficient mice, high-dose (5 mg/kg) PMX205 treatment significantly reduced the proportions of MDSCs in the colon and blood, and increased the proportions of CD8^+^ T cells in the blood, mucosa-associated lymphoid tissues and tumors (Figure 4C to E). In consistent with *C5ar1* deficiency, high but not low dose (1 mg/kg) of PMX205 treatment significantly attenuated the mouse conditions, as represented by improved body weight change (Figure S5A). In addition, high-dose PMX205 reduced the proportion of NK cells in the blood and spleen (Figure S5B) and the proportion of macrophages in the blood (Figure S5C), increased the proportion of the macrophages in the colon (Figure S5C) and the proportion of B cells in the spleen (Figure S5D), and had no effect on the proportion of CD4^+^ T cells (Figure S5E). Thus, these results indicate that the preventive effect of PMX205 resulted from C5aR1 inhibition, and that MDSCs and CD8^+^ T cells played pivotal roles in CRC development.

Moreover, 14 cytokines and chemokines that are intimately involved in preventing CRC initiation in *C5ar1*-deficient mice were also significantly altered upon PMX205 administration; including the decline of the proinflammatory cytokines TNF-α, IL-1α, IL-6, IL-1β and IL-11 (Figure 4F to J) and the chemokines CCL2, CXCL1 and CXCL5 (Figure 4L to N), and the elevation of the anti-inflammatory cytokine IL-10 (Figure 4K). The local levels of other cytokines and chemokines were shown in Figure S5F-G. Taken together, these findings further prove the prominent role of C5aR1 in initiating CRC and suggest that C5aR1 antagonists, such as PMX205, may hold great potential for preventing colorectal tumorigenesis.

### BM cells-derived C5aR1 initiates AOM/DSS-induced CRC

**Figure 5:**
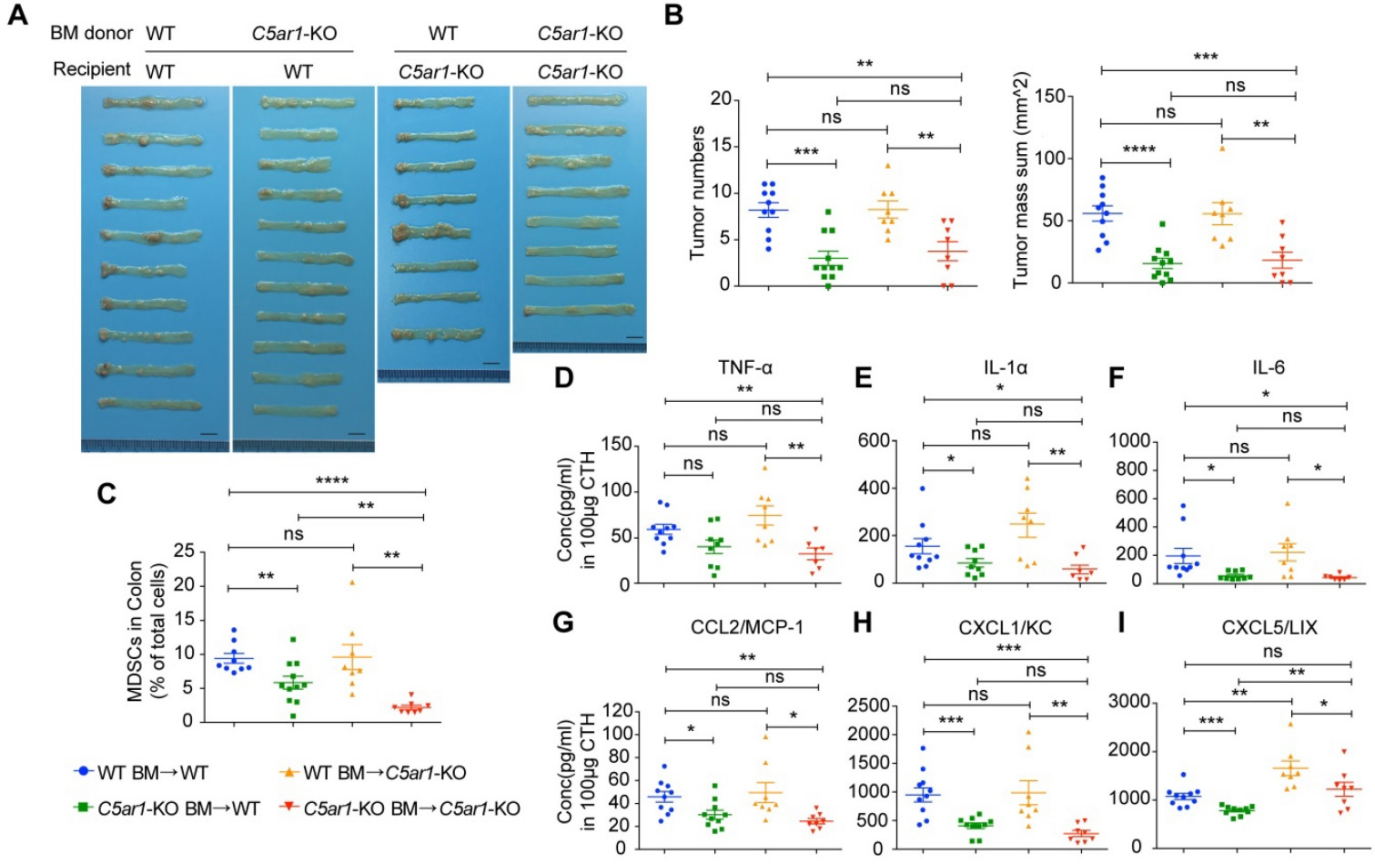
** BM transplantation reveals the sufficiency of C5aR1 expression in immune cells for initiating CRC.** (**A, B**) The effect of mutual BM transplantation between WT and *C5ar1*-deficient mice on AOM/DSS-induced colorectal tumorigenesis. Images of the colorectum (A) and quantitative analysis of tumor number and mass (B). Scale bar, 1 cm. (**C**) MDSC proportions in the colon. (**D-I**) Local levels of the proinflammatory cytokines TNF-α (D), IL-1α (E) and IL-6 (F) and the chemokines CCL2 (G), CXCL1 (H) and CXCL5 (I). Data are represented as mean ± SEM.; n≥8 in each group; ns, not significant; * *P*<0.05; ** *P*<0.01; *** *P*<0.001; and **** *P*<0.0001.

Considering that C5aR1 are expressed in immune cells and colonic epithelia, we next employed BM transplantation to determine the contribution of C5aR1 expression in immune cells and colorectal epithelial cells to CRC initiation. BM cells were mutually transplanted between WT and* C5ar1*-deficient mice after radiation treatment, and the established chimeric mice were confirmed by detecting C5aR1 expression in BM cells (Figure S6A). We unexpectedly found that *C5ar1*-deficient mice exhibited much worse conditions than WT mice after radiation treatment; therefore, *C5ar1*-deficient recipient mice displayed lower survival rates than WT recipients although there was no statistical significance (Figure S6B), which appeared not to correlate with CRC development after AOM/DSS induction. Mice transplanted with *C5ar1*-deficient donor BM cells developed significantly fewer tumors than those receiving WT donor BM cells (Figure 5A-B), and mouse body weight change showed a similar pattern (Figure S6C-D). Importantly, there were no significant differences in tumor numbers or size between different recipients subjected to injection of the same donor BM cells (Figure 5A-B), indicating that BM cells-derived C5aR1 determines the initiation of CRC.

In addition, mice receiving *C5ar1*-deficient donor BM cells displayed a significantly lower proportion of MDSCs in the colon than the corresponding mice transplanted with WT donor BM cells, and *C5ar1*-deficient recipients showed a much lower proportion of MDSCs in the colon than WT recipients (Figure 5C). However, the proportions of MDSCs in the blood and spleen and the proportions of macrophages, CD8^+^ T cells, CD4^+^ T cells, NK cells and B cells in the indicated tissues (except for B cells in the spleen) failed to correlate well with the pattern of CRC development in the four groups of chimeric mice (Figure S7A to F). Therefore, local MDSCs appear to be a determinant cell type for AOM/DSS-induced CRC initiation.

Further, the results of local cytokines/chemokines measurements in these chimeric mice after AOM/DSS treatment also supported the critical role of C5aR1 expression in immune cells. The local levels of the important cytokines, TNF-α, IL-1α and IL-6 (Figure 5D to F) and chemokines CCL2, CXCL1 and CXCL5 (Figure 5G to I), all of which were previously shown to be highly involved in preventing CRC development in *C5ar1*-deficient mice, decreased significantly (except for TNF-α in WT recipients) in mice receiving *C5ar1*-deficient donor BM cells compared with mice transplanted with WT donor BM cells. The local levels of other detectable cytokines/chemokines failed to display close correlations with CRC development in the four groups of chimeric mice (Figure S7G-H). These data indicate that C5aR1 on immune cells is crucial for colorectal tumorigenesis.

## Discussion

Although cancer incidence and cancer-related deaths have gradually decreased in the US since the 1990s 1, cancer still has major impacts on public health around the world. However, there is a lack of effective strategies for cancer prevention, including CRC, due to the obscure mechanisms of tumorigenesis and the absence of precise drug targets.

A number of studies have demonstrated the important roles of complement activation in CRC growth and metastasis using syngeneic or xenograft mouse models 19, 21, 22, 33, which was summarized in an elegant review 14; however, there are limited investigations to explore the association of complement activation with tumor initiation including CRC tumorigenesis 10-14. A previous report showed that the deficiency of *C3*, *C5* or *C5ar1* significantly induced tumor repression in an AOM/DSS-induced CRC tumorigenesis model, which may result from the suppressed local IL-1β/IL-17A axis in neutrophils 24. Herein, using a similar model, we revealed that complement C5a/C5aR1, but not C3, may be a master regulator to initiate colorectal tumorigenesis by modulating versatile immune responses. This discrepancy may be attributed to the different genetic background of the mouse strains and the severity of AOM/DSS induction, in which we optimized to induce a much more severe CRC determined by aggravated disease activity, larger tumor number and size (Figure 1E- F; and Figure S1B-C).

C5 could be cleaved and activated by a series of protease enzymes, including plasmin, trypsin, cathepsins and thrombin 14, 17, 34-36. A recent report revealed that in a C3-independent manner, C5 could be cleaved by plasmin that is activated by urokinase (uPA)-expressing macrophages, thus fostering mouse squamous carcinogenesis 17. Herein, we report that *C5*-, but not *C3*-deficient mice showed the dramatically dampened colorectal tumorigenesis via C5a/C5aR1 signaling, indicating C5 activation through a non-canonical, cascade-independent mechanism. Interestingly, recent evidence shows that thrombin is produced in tumors and has potent pro-tumor activity 37; furthermore, thrombin supports early events coupled to inflammation-mediated tumorigenesis in CAC 38. Therefore, it is tempting to propose that C5 may be cleaved and activated by thrombin 34, 35 in AOM/DSS-induced CAC model, which needs further investigation.

C5aR2, probably as a decoy receptor of C5a, may impair the pro-inflammatory response of C5a by regulating the bioavailability of C5a 39. It may also negatively modulate C5aR1-mediated signal transduction 28, 40. However, C5aR2 instead displays a pro-inflammatory role in several autoimmune diseases, resulting in an enigmatic and controversial role 41. In consistent with a previous report in a melanoma bearing murine model 42, we showed that in contrast to C5aR1, C5aR2 deficiency accelerated tumor development, suggesting an anti-inflammatory role of C5aR2 in AOM/DSS-induced CRC tumorigenesis. The result may also explain the observation that tumor burden in *C5-*deficient mice is greater than in *C5ar1-*deficient mice.

MDSCs have long been suspected to be strongly associated with tumor development at diverse stages via crosstalk with other immune cells, including CD8^+^ T cells, thus constructing a strong immunosuppressive environment 15, 43. In AOM/DSS-induced CRC mice, we found that the reduction in MDSCs and concomitant increase in CD8^+^ T cells in the tumor, blood and/or spleen correlated with the preventive effects of *C5* and *C5ar1* deficiency, and a C5aR1 antagonist on CRC development. Moreover, MDSC proportions are highly inversely correlated with CD8^+^ T cell proportions in the blood and spleen. Furthermore, in BM chimeric mice, *C5ar1*-deficient donors resulted in a lower infiltration of MDSCs into the colon than WT donors, thus retarding CRC development. These findings suggest that local MDSCs may create a strong immunosuppressive environment in which the inhibition of CD8^+^ T cell propagation plays a critical role in initiating CRC.

Multiple pro- and anti-inflammatory cytokines have been recognized to participate in CRC, peculiarly CAC development, via various mechanisms. Proinflammatory cytokines, such as TNF-α, IL-1α/β, IL-6, IL-17A, and IL-11, and their downstream transcription factors, including NF-κB and STAT3, construct an immunosuppressive stroma that addicts tumor cells; therefore, these factors are emerging as potential targets for CRC therapy^2^, ^31^, ^32^, ^44^. In contrast, anti-inflammatory cytokines, such as IL-9, IL-23, IL-27, and especially IL-10, play the opposite role, thus retarding CRC tumorigenesis 2, 31, 32. IL-10-deficient mice develop spontaneous severe colitis and consequently CRC, which can be attenuated by IL-10 administration and antibiotic treatment 45. In addition, chemokines, at least in part, drive CRC development by recruiting leukocytes from the circulatory system to the local inflammatory microenvironment 46. The local levels of proinflammatory chemokines such as CCL2, CCL3, CCL4, and CCL5 and proangiogenic chemokines such as CXCL1, CXCL5, and CXCL8 are elevated in human colon tumor tissues compared to those in paired normal tissues 47, 48. CCL2, a potent macrophage chemoattractant, is especially strongly associated with colorectal tumorigenesis by fostering colonic polymorphonuclear MDSC accumulation and the resultant inhibition of T cells via ROS and NO 3. The CCR4 ligands CCL17 and CCL22, which are especially polarized to Th2 cells and Tregs, are also elevated in CRC tissues 49. Moreover, CXCR2-expressing MDSCs have been demonstrated to be essential for promoting CAC accompanied by increased expression levels of CXCR2 and its ligands, including CXCL1, CXCL2, and CXCL5 4, 50. Furthermore, in chimeric mice subjected to AOM/DSS treatment, *C5ar1*-deficient but not WT BM donors significantly reduced the levels of TNF-α, IL-1α, IL-6, CCL2, CXCL1, and CXCL5, indicating that these mediators are mainly produced by immune cells. It has been reported that intracellular C5aR1 can promote the synthesis and maturation of IL-1β during Th1 cell activation 28. However, we found that a single-allele deficiency of *C5* or especially *C5ar1* potently decreased the levels of proinflammatory cytokines (TNF-α, IL-1α/β, IL-6, IL-17A, and IL-11) and chemokines (CCL2, CCL17, CXCL1, and CXCL5) and elevated the levels of anti-inflammatory cytokines (IL-23, IL-9, IL-27, and especially IL-10). All of these factors have been previously demonstrated to be crucial for CRC development, and most of these effects were confirmed by C5aR1 antagonist treatment. Therefore, these findings render C5aR1 central to cytokines/chemokines regulation and subsequent immunosuppressive cells recruitment during CRC development.

## Conclusion

**Figure 6:**
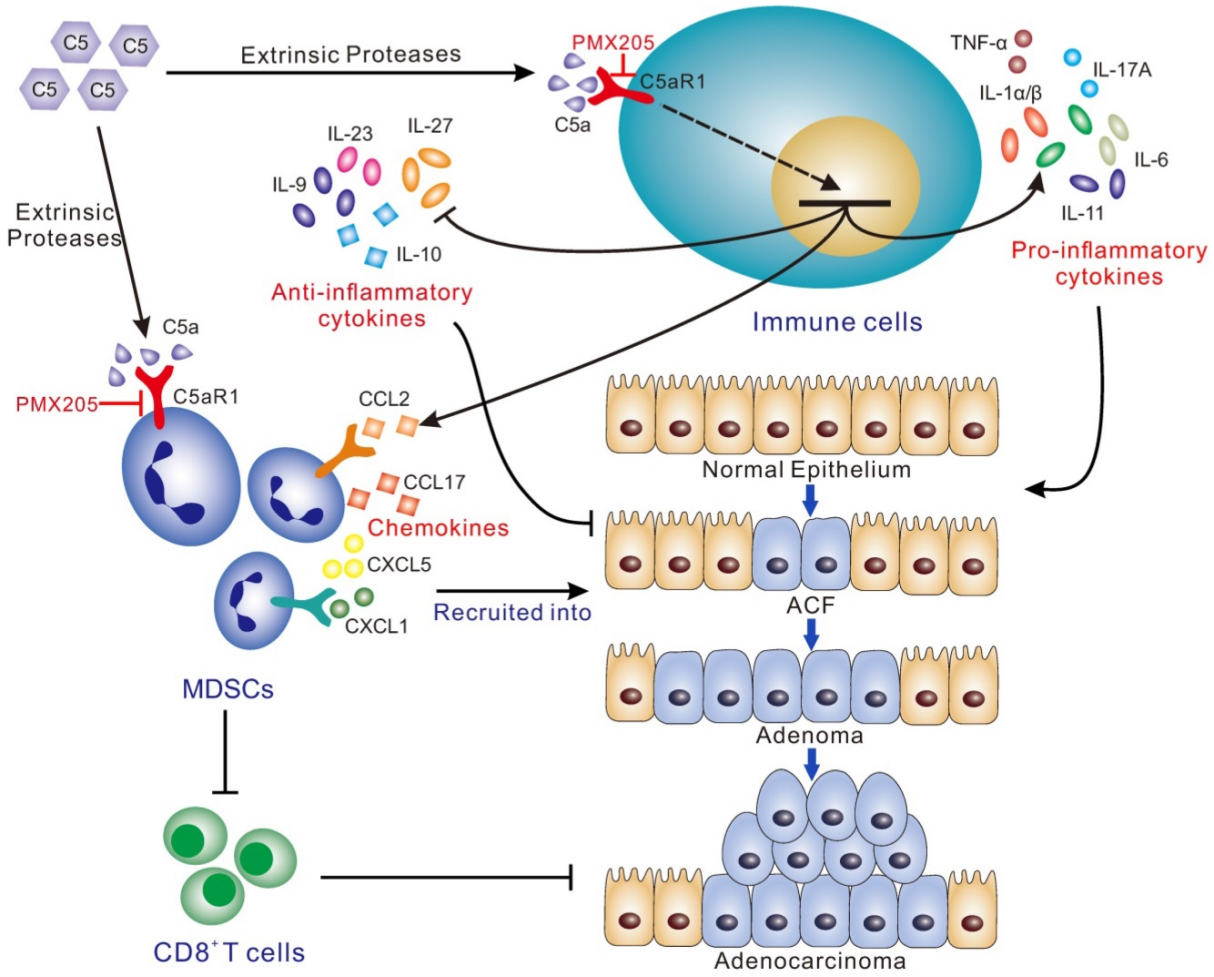
** Schematic for the mechanisms by which C5a/C5aR1 signaling initiates colorectal tumorigenesis by modulating versatile immune responses.** Complement C5a/C5aR1 signaling, independent of C3 activation, recruits MDSCs into the inflamed colorectal tissues to impair CD8^+^ T cells, and modulates the production of a variety of cytokines/chemokines (IL-1, IL-6, IL-11, IL-17A, TNF-α, IL-9, IL-10, IL-23, IL-27, CCL2, CCL17, CXCL1/5), thus fostering AOM/DSS-induced colorectal tumorigenesis. C5aR1 inhibition by PMX205 impedes CRC tumorigenesis. ACF, aberrant crypt foci.

Together, our study, specifically focusing on the implication of complement in colorectal tumorigenesis, revealed that C5aR1 is a master regulator with pleiotropic effects that initiates CRC by modulating a tumor-promoting immune response; thus, C5aR1 may exert as a promising potential target for CRC prevention (Figure 6).

## Supplementary Material

Supplementary figures and tables.

## Abbreviations

AOM: azoxymethane
BM: bone marrow
CAC: colitis-associated cancer
CRC: colorectal cancer
CTH: colon tissue homogenate
DSS: dextran sulfate sodium
ELISA: enzyme-linked immunosorbent assay
H&E: hematoxylin and eosin
IBD: inflammatory bowel disease
IHC: immunohistochemistry
MAC: membrane attack complex
MALT: mucosa-associated lymphoid tissue
MDSC: myeloid-derived suppressor cell
NK: natural killer
TAM: tumor-associated macrophage
TIL: tumor-infiltrating lymphocyte
WT: wild-type

## References

[1] Siegel RL, Miller KD, Jemal A (2020). Cancer statistics, 2020. CA Cancer J Clin.

[2] Terzic J, Grivennikov S, Karin E, Karin M (2010). Inflammation and colon cancer. Gastroenterology.

[3] Chun E, Lavoie S, Michaud M, Gallini CA, Kim J, Soucy G (2015). CCL2 promotes colorectal carcinogenesis by enhancing polymorphonuclear myeloid-derived suppressor cell population and function. Cell Rep.

[4] Katoh H, Wang D, Daikoku T, Sun H, Dey SK, Dubois RN (2013). CXCR2-expressing myeloid-derived suppressor cells are essential to promote colitis-associated tumorigenesis. Cancer Cell.

[5] Grivennikov S, Karin E, Terzic J, Mucida D, Yu GY, Vallabhapurapu S (2009). IL-6 and Stat3 are required for survival of intestinal epithelial cells and development of colitis-associated cancer. Cancer Cell.

[6] Popivanova BK, Kitamura K, Wu Y, Kondo T, Kagaya T, Kaneko S (2008). Blocking TNF-alpha in mice reduces colorectal carcinogenesis associated with chronic colitis. J Clin Invest.

[7] Ricklin D, Hajishengallis G, Yang K, Lambris JD (2010). Complement: A key system for immune surveillance and homeostasis. Nat Immunol.

[8] Merle NS, Church SE, Fremeaux-Bacchi V, Roumenina LT (2015). Complement system part I - molecular mechanisms of activation and regulation. Front Immunol.

[9] Merle NS, Noe R, Halbwachs-Mecarelli L, Fremeaux-Bacchi V, Roumenina LT (2015). Complement system part II: Role in immunity. Front Immunol.

[10] Afshar-Kharghan V (2017). The role of the complement system in cancer. J Clin Invest.

[11] Pio R, Corrales L, Lambris JD (2014). The role of complement in tumor growth. Adv Exp Med Biol.

[12] Pio R, Ajona D, Ortiz-Espinosa S, Mantovani A, Lambris JD (2019). Complementing the cancer-immunity cycle. Front Immunol.

[13] Reis ES, Mastellos DC, Ricklin D, Mantovani A, Lambris JD (2018). Complement in cancer: Untangling an intricate relationship. Nat Rev Immunol.

[14] Roumenina LT, Daugan MV, Petitprez F, Sautes-Fridman C, Fridman WH (2019). Context-dependent roles of complement in cancer. Nat Rev Cancer.

[15] Markiewski MM, DeAngelis RA, Benencia F, Ricklin-Lichtsteiner SK, Koutoulaki A, Gerard C (2008). Modulation of the antitumor immune response by complement. Nat Immunol.

[16] Bonavita E, Gentile S, Rubino M, Maina V, Papait R, Kunderfranco P (2015). PTX3 is an extrinsic oncosuppressor regulating complement-dependent inflammation in cancer. Cell.

[17] Medler TR, Murugan D, Horton W, Kumar S, Cotechini T, Forsyth AM (2018). Complement C5a fosters squamous carcinogenesis and limits T cell response to chemotherapy. Cancer Cell.

[18] Habermann JK, Roblick UJ, Luke BT, Prieto DA, Finlay WJ, Podust VN (2006). Increased serum levels of complement C3a anaphylatoxin indicate the presence of colorectal tumors. Gastroenterology.

[19] Downs-Canner S, Magge D, Ravindranathan R, O'Malley ME, Francis L, Liu Z (2016). Complement inhibition: A novel form of immunotherapy for colon cancer. Ann Surg Oncol.

[20] Nitta H, Wada Y, Kawano Y, Murakami Y, Irie A, Taniguchi K (2013). Enhancement of human cancer cell motility and invasiveness by anaphylatoxin C5a via aberrantly expressed C5a receptor (CD88). Clin Cancer Res.

[21] Piao C, Cai L, Qiu S, Jia L, Song W, Du J (2015). Complement 5a enhances hepatic metastases of colon cancer via monocyte chemoattractant protein-1-mediated inflammatory cell infiltration. J Biol Chem.

[22] Piao C, Zhang WM, Li TT, Zhang CC, Qiu S, Liu Y (2018). Complement 5a stimulates macrophage polarization and contributes to tumor metastases of colon cancer. Exp Cell Res.

[23] Doerner SK, Reis ES, Leung ES, Ko JS, Heaney JD, Berger NA (2016). High-fat diet-induced complement activation mediates intestinal inflammation and neoplasia, independent of obesity. Mol Cancer Res.

[24] Ning C, Li YY, Wang Y, Han GC, Wang RX, Xiao H (2015). Complement activation promotes colitis-associated carcinogenesis through activating intestinal IL-1beta/IL-17A axis. Mucosal Immunol.

[25] Wang Q, Wang N, Zhang X, Hu W (2015). A simple PCR-based method for the rapid genotyping of inherited fifth complement component (C5)-deficient mice. Exp Anim.

[26] Neufert C, Becker C, Neurath MF (2007). An inducible mouse model of colon carcinogenesis for the analysis of sporadic and inflammation-driven tumor progression. Nat Protoc.

[27] Lin F, Spencer D, Hatala DA, Levine AD, Medof ME (2004). Decay-accelerating factor deficiency increases susceptibility to dextran sulfate sodium-induced colitis: Role for complement in inflammatory bowel disease. J Immunol.

[28] Arbore G, West EE, Spolski R, Robertson AA, Klos A, Rheinheimer C (2016). T helper 1 immunity requires complement-driven NLRP3 inflammasome activity in CD4(+) T cells. Science.

[29] Colligan SH, Tzetzo SL, Abrams SI (2019). Myeloid-driven mechanisms as barriers to antitumor CD8(+) T cell activity. Mol Immunol.

[30] Veglia F, Perego M, Gabrilovich D (2018). Myeloid-derived suppressor cells coming of age. Nat Immunol.

[31] Klampfer L (2011). Cytokines, inflammation and colon cancer. Curr Cancer Drug Targets.

[32] Francescone R, Hou V, Grivennikov SI (2015). Cytokines, IBD, and colitis-associated cancer. Inflamm Bowel Dis.

[33] Surace L, Lysenko V, Fontana AO, Cecconi V, Janssen H, Bicvic A (2015). Complement is a central mediator of radiotherapy-induced tumor-specific immunity and clinical response. Immunity.

[34] Huber-Lang M, Sarma JV, Zetoune FS, Rittirsch D, Neff TA, McGuire SR (2006). Generation of C5a in the absence of C3: A new complement activation pathway. Nat Med.

[35] Krisinger MJ, Goebeler V, Lu Z, Meixner SC, Myles T, Pryzdial EL (2012). Thrombin generates previously unidentified C5 products that support the terminal complement activation pathway. Blood.

[36] Amara U, Flierl MA, Rittirsch D, Klos A, Chen H, Acker B (2010). Molecular intercommunication between the complement and coagulation systems. J Immunol.

[37] Reddel CJ, Tan CW, Chen VM (2019). Thrombin generation and cancer: Contributors and consequences. Cancers (Basel).

[38] Turpin B, Miller W, Rosenfeldt L, Kombrinck K, Flick MJ, Steinbrecher KA (2014). Thrombin drives tumorigenesis in colitis-associated colon cancer. Cancer Res.

[39] Gerard NP, Lu B, Liu P, Craig S, Fujiwara Y, Okinaga S (2005). An anti-inflammatory function for the complement anaphylatoxin C5a-binding protein, C5L2. J Biol Chem.

[40] Bamberg CE, Mackay CR, Lee H, Zahra D, Jackson J, Lim YS (2010). The C5a receptor (C5aR) C5L2 is a modulator of C5aR-mediated signal transduction. J Biol Chem.

[41] Woodruff TM, Nandakumar KS, Tedesco F (2011). Inhibiting the C5-C5a receptor axis. Mol Immunol.

[42] Nabizadeh JA, Manthey HD, Panagides N, Steyn FJ, Lee JD, Li XX C5a receptors C5aR1 and C5aR2 mediate opposing pathologies in a mouse model of melanoma. FASEB J. 2019: fj201800980RR.

[43] Ostrand-Rosenberg S, Sinha P, Beury DW, Clements VK (2012). Cross-talk between myeloid-derived suppressor cells (MDSC), macrophages, and dendritic cells enhances tumor-induced immune suppression. Semin Cancer Biol.

[44] Liu YJ, Tang B, Wang FC, Tang L, Lei YY, Luo Y (2020). Parthenolide ameliorates colon inflammation through regulating Treg/Th17 balance in a gut microbiota-dependent manner. Theranostics.

[45] Berg DJ, Davidson N, Kuhn R, Muller W, Menon S, Holland G (1996). Enterocolitis and colon cancer in interleukin-10-deficient mice are associated with aberrant cytokine production and CD4(+) Th1-like responses. J Clin Invest.

[46] Wang D, Dubois RN, Richmond A (2009). The role of chemokines in intestinal inflammation and cancer. Curr Opin Pharmacol.

[47] Baier PK, Eggstein S, Wolff-Vorbeck G, Baumgartner U, Hopt UT (2005). Chemokines in human colorectal carcinoma. Anticancer Res.

[48] Ha H, Debnath B, Neamati N (2017). Role of the CXCL8-CXCR1/2 axis in cancer and inflammatory diseases. Theranostics.

[49] Al-haidari AA, Syk I, Jirstrom K, Thorlacius H (2013). CCR4 mediates CCL17 (TARC)-induced migration of human colon cancer cells via RhoA/Rho-kinase signaling. Int J Colorectal Dis.

[50] Liu ZY, Zheng M, Li YM, Fan XY, Wang JC, Li ZC (2019). RIP3 promotes colitis-associated colorectal cancer by controlling tumor cell proliferation and CXCL1-induced immune suppression. Theranostics.

