# Supplementary material for: C5aR1 is a master regulator in Colorectal Tumorigenesis via Immune modulation
Source: Theranostics. 2020 Jul 9;10(19):8619–32. doi: 10.7150/thno.45058 (PMC7392014; doi:10.7150/thno.45058)
Supplement: Supplementary file 1 — Supplementary figures and tables. [file thnov10p8619s1.pdf]

Supplementary figures and figure legends

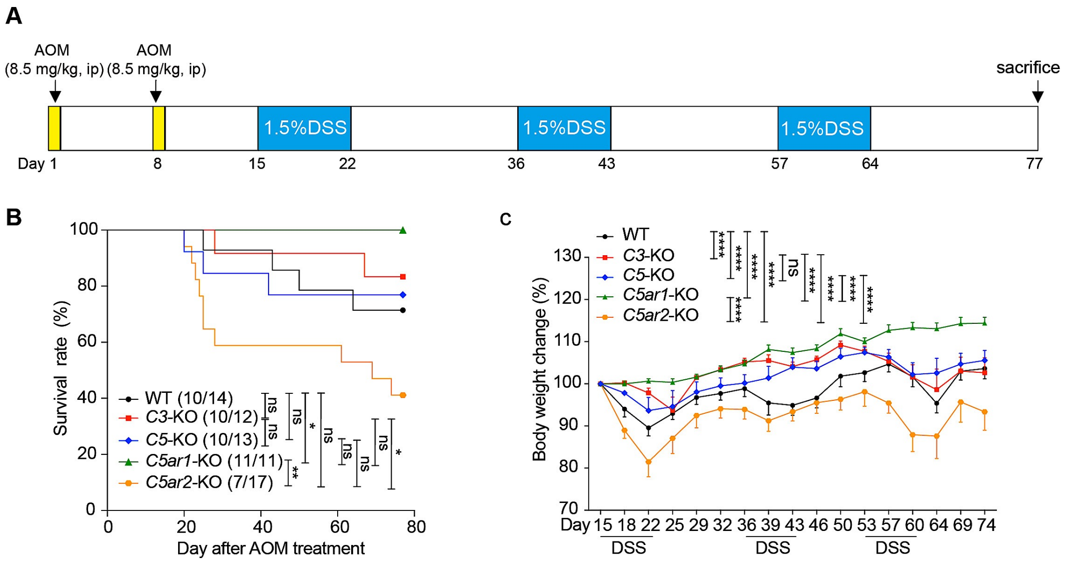

Figure S1. Clinical effect of AOM/DSS exposure in WT and *C3*-, *C5*-, *C5ar1*- or *C5ar2*-deficient mice. (**A**) Schematic overview of mice treated with AOM and DSS. (**B, C**) The dynamic survival rate (B) and body weight change (C) over 77-day exposure to AOM/DSS. The numbers of mice at the starting point (denominator) and endpoint (numerator) are shown in brackets (B). Data are represented as mean ± SEM; n=7-14 per group; ns, not significant; * $P<0.05$; ** $P<0.01$; and **** $P<0.0001$.

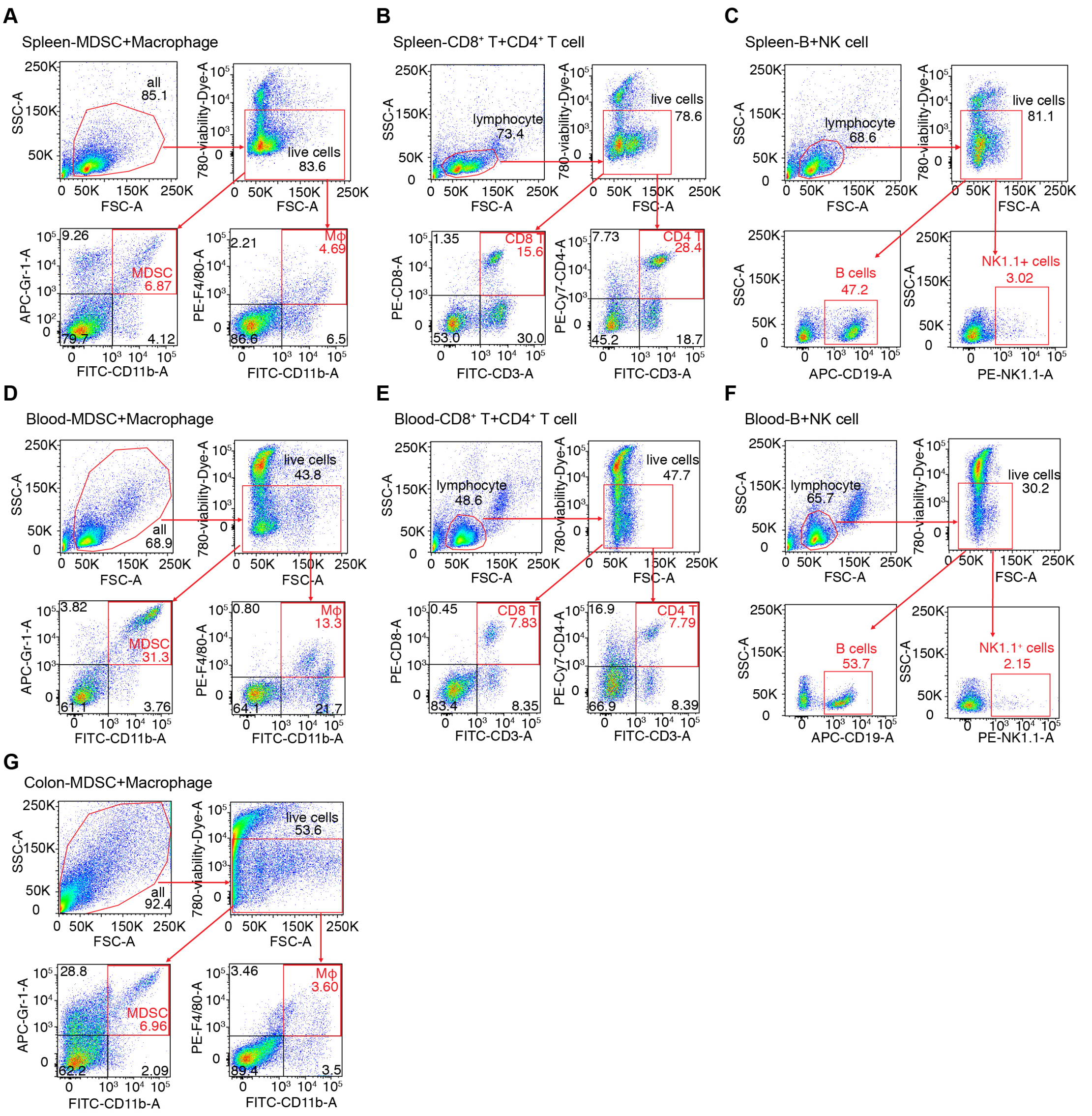

Figure S2. Gating strategy for immunocyte subsets characterized by flow cytometry. **(A-G)** The gating strategies to identify MDSC (CD11b$^+$Gr-1$^+$), Macrophage (CD11b$^+$F4/80$^+$), CD8$^+$ T cell (CD3$^+$CD8$^+$), CD4$^+$ T cell (CD3$^+$CD4$^+$), B cell (CD19$^+$) and NK cell (NK1.1$^+$) in spleen (A-C), blood (D-F) and colon tissues (G). FSC, forward scatter; SSC, side scatter.

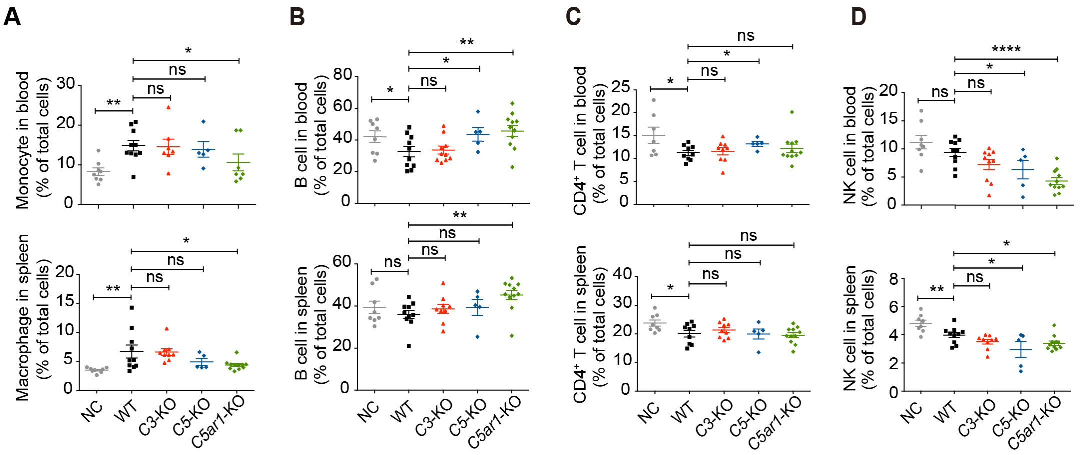

Figure S3. The profiles of immune cells in WT and *C3*-, *C5*-, or *C5ar1*-deficient mice treated with AOM/DSS. (**A-D**) The proportions of Monocyte/Macrophage (A), B cell (B), $CD4^+$ T cell (C), and NK cell (D) in the blood (upper panel) and spleen (lower panel) of WT and *C3*-, *C5-, or C5ar1*-deficient mice upon AOM/DSS treatment. The negative control (NC) refers to WT mice without AOM/DSS exposure. Data are represented as mean ± SEM; n≥5 in each group; ns, not significant; * *P*<0.05; ***P*<0.01; and **** *P*<0.0001.

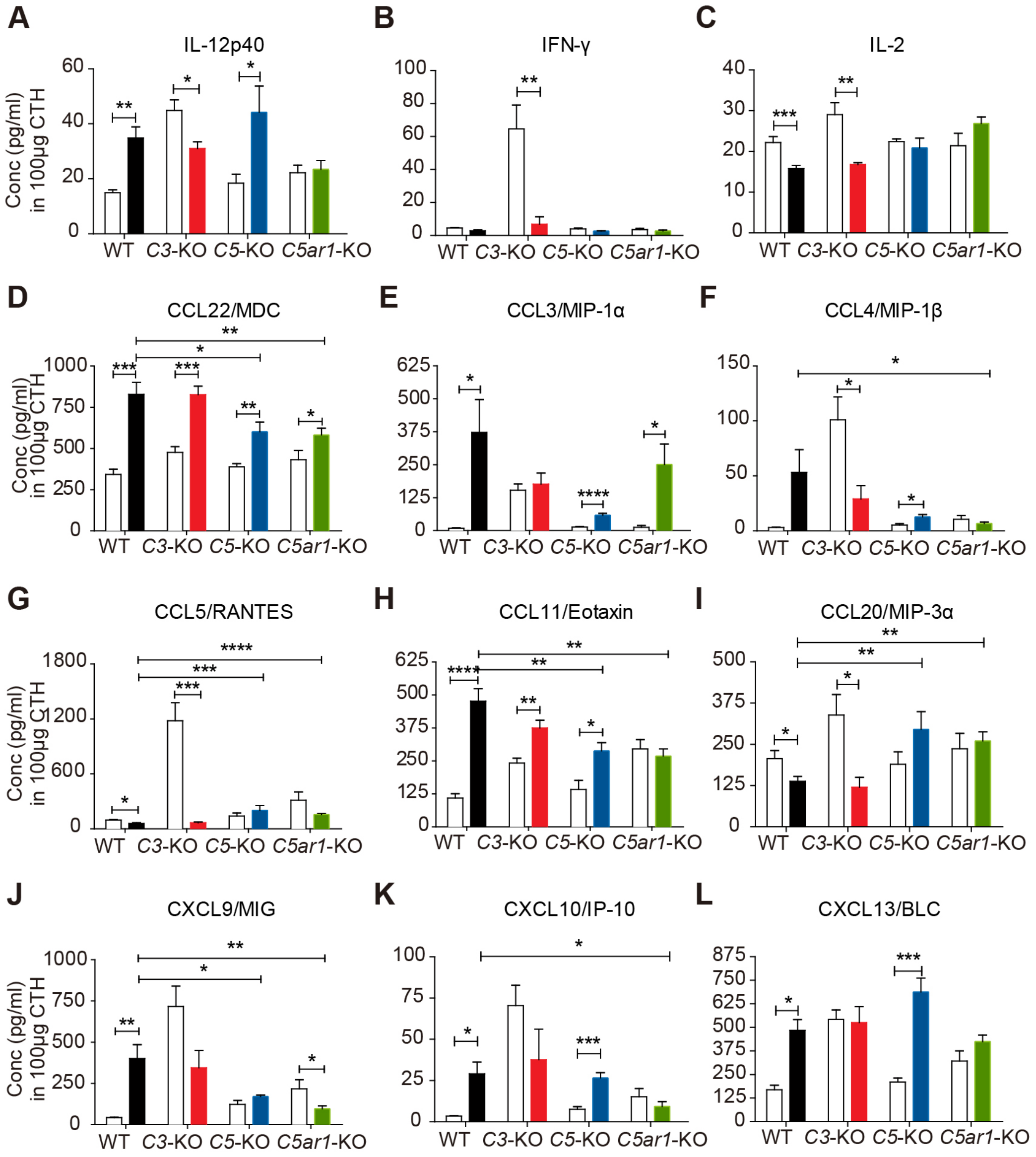

Figure S4. The profiles of multiple cytokines/chemokines in WT and *C3*-, *C5*-, or *C5ar1*-deficient mice upon AOM/DSS treatment. (**A-C**) The effect of *C3*, *C5*, or *C5ar1* deficiency on the local levels of the indicated cytokines IL-12p40 (A), IFN-γ (B) and IL-2 (C). (**D-L**) Local levels of the indicated chemokines CCL22 (D), CCL3 (E), CCL4 (F), CCL5 (G), CCL11 (H), CCL20 (I), CXCL9 (J), CXCL10 (K) and CXCL13 (L) in AOM/DSS-treated mice. The undetectable cytokines or chemokines via LEGENDplex include GM-CSF, IFN-β, IL-3, IL-4, IL-5, IL-7, IL-12p70, IL-13, IL-

17F, IL-21, IL-22, IL-33, and TSLP. Data are represented as mean ± SEM; n≥5 in each group; ns, not significant; * *P*<0.05; ** *P*<0.01; *** *P*<0.001; and **** *P*<0.0001.

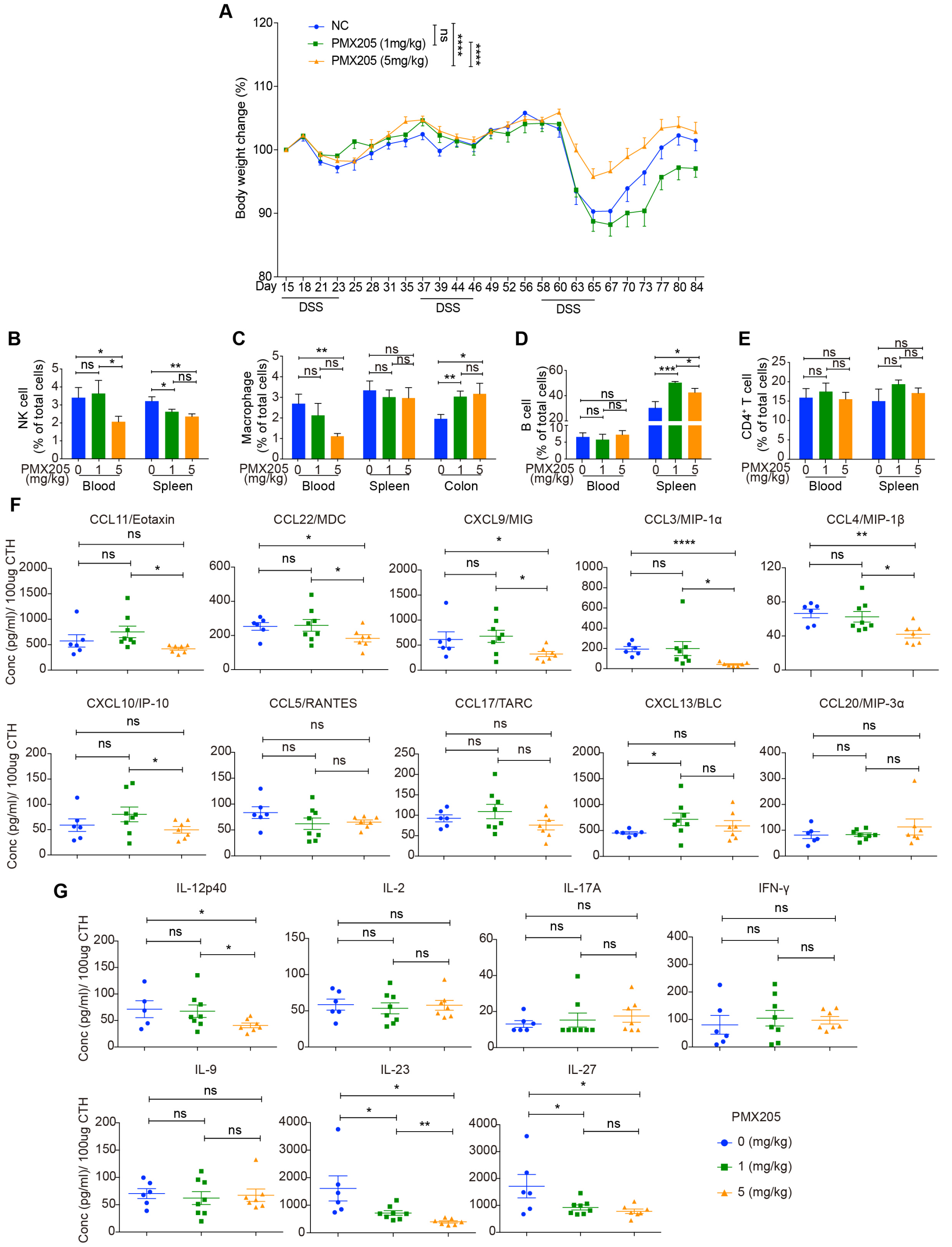

Figure S5. Clinical effect and immune response in AOM/DSS-induced CRC mice treated with the C5aR1 antagonist PMX205. (**A**) The dynamic body weight change in mice upon AOM/DSS treatment with or without PMX205 administration. (**B-E**) The proportions of NK cell (B), Macrophage (C), B cell (D), and CD4^{+} T cell (E) in the

indicated tissues of AOM/DSS-treated mice with or without PMX205 administration. (**F-G**) The effect of PMX205 on the local levels of the indicated chemokines (F) and cytokines (G). Undetectable cytokines or chemokines via LEGENDplex are not shown. Data are represented as mean± SEM; n≥5 in each group; ns, not significant; * $P<0.05$; ** $P<0.01$; *** $P<0.001$; and **** $P<0.0001$.

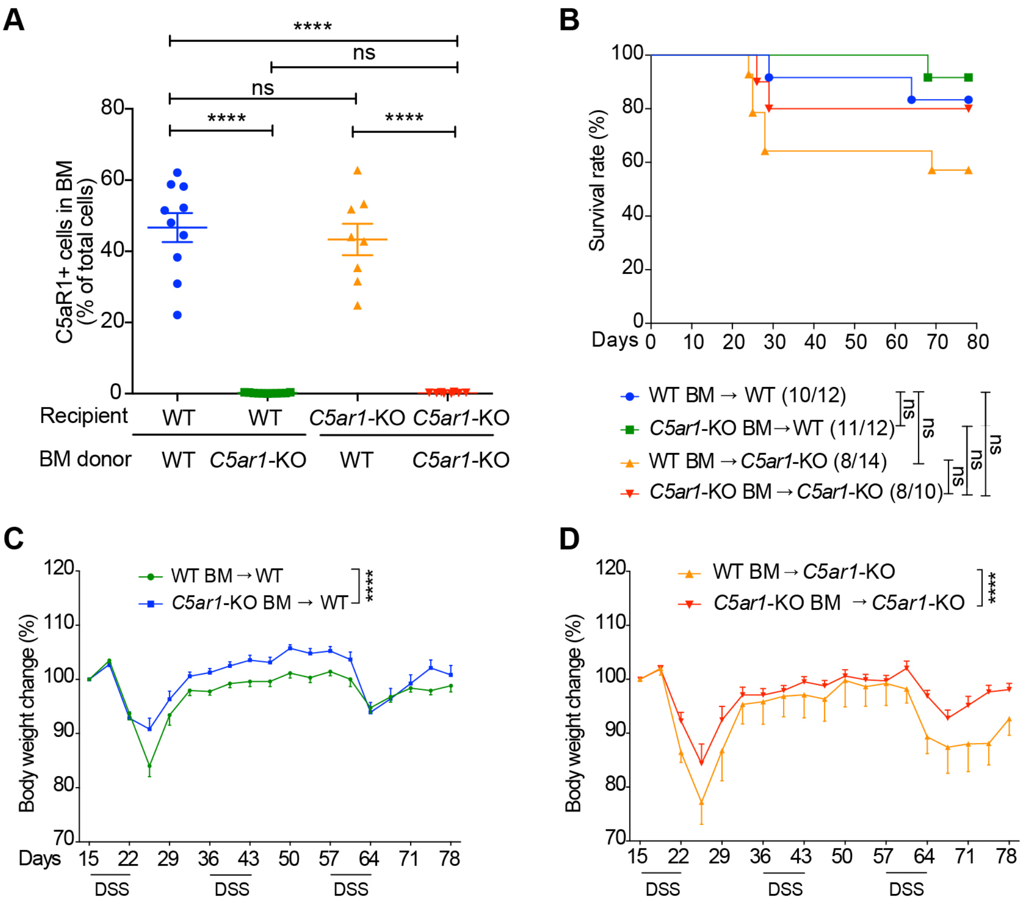

Figure S6. Clinical effect of BM transplantation in chimeric mice with AOM/DSS exposure. (**A**) C5aR1 expression in immune cells from the BM of chimeric mice. n≥8. (**B**) Survival rate of chimeric mice upon AOM/DSS treatment. The numbers of mice at the starting point (denominator) and endpoint (numerator) are shown in brackets. (**C, D**) The dynamic body weight change of chimeric mice after exposure to AOM/DSS. Data are represented as mean± SEM; ns, not significant; **** *P*<0.0001.

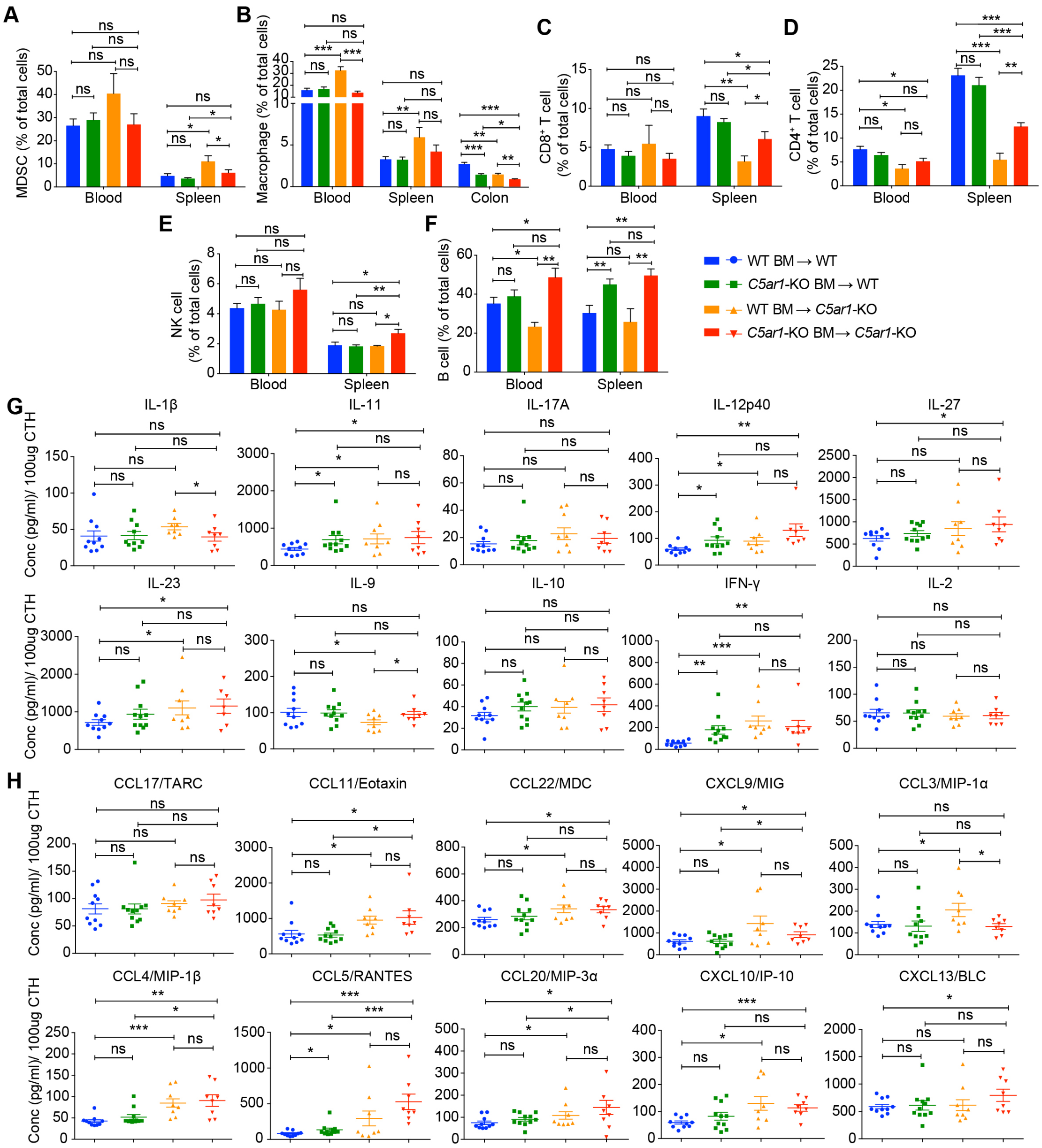

Figure S7. Immune response in BM chimeric mice with AOM/DSS treatment. (**A-F**) The proportions of MDSC (A), Macrophage (B), CD8$^+$ T cell (C), CD4$^+$ T cell (D), NK cell (E), and B cell (F) in the indicated tissues of chimeric mice upon AOM/DSS treatment. (**G-H**) The effect of BM transplantation on the local levels of the indicated cytokines (G) and chemokines (H). Undetectable cytokines or chemokines via LEGENDplex are not shown. Data are represented as mean± SEM; n≥8 in each group; ns, not significant; * *P*<0.05; ** *P*<0.01; and *** *P*<0.001.

Table S1. The commercial antibodies used in this study.

Antibodies	Source	Identifier
Mouse Complement Component C3d	R&D Systems	Cat# AF2655
Ki67	Abcam	Cat# ab15580
CD8 alpha	Abcam	Cat# ab217344
FITC anti-mouse CD11b	Biolegend	Cat# 101206
FITC anti-mouse CD3ε	Biolegend	Cat# 100306
PE anti-mouse CD8a	Biolegend	Cat# 100708
PE anti-mouse NK1.1	Biolegend	Cat# 108708
PE anti-mouse CD88	Biolegend	Cat# 135805
APC anti-mouse F4/80	Biolegend	Cat# 123116
APC anti-mouse CD19	Biolegend	Cat# 115512
APC anti-mouse Gr-1	Biolegend	Cat# 108412
PE/Cy7 anti-mouse CD4	Biolegend	Cat# 100528
Purified anti-mouse CD16/32	Biolegend	Cat# 101302
Rabbit Anti-Goat IgG H&L (HRP)	Abcam	Cat# ab6741
Goat Anti-Rabbit IgG H&L (HRP)	Abcam	Cat# ab97051

Table S2. Basic features of colorectal cancer patients from TCGA*.

Variable	N	%
**Age**		
<60	130	35.71
≥60	234	64.29
**Sex**		
Male	202	55.49
Female	162	44.51
**Stage**		
I	56	15.38
II	129	35.44
III	110	30.22
IV	50	13.74
Unknown	19	5.22
**Histology**		
Adenocarcinoma	318	87.36
Mucinous Adenocarcinoma	41	11.26
Unknown	5	1.37

* Clinical data were downloaded from cBioPortal.
